# Genotyping of *Mycobacterium tuberculosis *clinical isolates in two cities of Turkey: Description of a new family of genotypes that is phylogeographically specific for Asia Minor

**DOI:** 10.1186/1471-2180-5-44

**Published:** 2005-07-26

**Authors:** Thierry Zozio, Caroline Allix, Selami Gunal, Zeynep Saribas, Alpaslan Alp, Riza Durmaz, Maryse Fauville-Dufaux, Nalin Rastogi, Christophe Sola

**Affiliations:** 1 Unité de la Tuberculose et des Mycobactéries, Institut Pasteur de Guadeloupe; 2 Laboratoire de la Tuberculose, Institut Pasteur de Bruxelles; 3 Department of Clinical Microbiology, Faculty of Medicine, Inonu University, Malatya, Turkey; 4 Department of Microbiology and Clinical Microbiology, Faculty of Medicine, Hacettepe University, Ankara, Turkey

## Abstract

**Background:**

Population-based bacterial genetics using repeated DNA loci is an efficient approach to study the biodiversity and phylogeographical structure of human pathogens, such as *Mycobacterium tuberculosis*, the agent of tuberculosis. Indeed large genetic diversity databases are available for this pathogen and are regularly updated. No population-based polymorphism data were yet available for *M. tuberculosis *in Turkey, at the crossroads of Eurasia.

**Results:**

A total of 245 DNAs from *Mycobacterium tuberculosis *clinical isolates from tuberculosis patients residing in Turkey (Malatya n = 147 or Ankara n = 98) were genotyped by spoligotyping, a high-throughput genotyping method based on the polymorphism of the Direct Repeat locus. Thirty-three spoligotyping-defined clusters including 206 patients and 39 unique patterns were found. The ST41 cluster, as designated according to the international SpolDB3 database project, represented one fourth and when gathered to three genotypes, ST53, ST50 and ST284, one half of all the isolates. Out of 34 clinical isolates harboring ST41 which were further genotyped by IS*6110 *and by MIRU-VNTR typing, a typical 2-copy IS*6110*-RFLP pattern and a "215125113322" MIRU-VNTR pattern were observed among 21 clinical isolates. Further search in various databases confirms the likely Turkish-phylogeographical specificity of this clonal complex.

**Conclusion:**

We described a new phylogeographically-specific clone of *M. tuberculosis*, designated LAM7-TUR. Further investigations to assess its frequency within all regions of Turkey and its phylogeographical origin and phylogenetic position within the global *M. tuberculosis *phylogenetic tree will shed new light on its endemicity in Asia Minor.

## Background

Turkey is a large and densely populated country (area.: 780 576 km^2^, population: around 71 millions). In Turkey, tuberculosis remains an important public health concern with a case notification rate of 26.2/100.000 inhabitants in 2002 [1]. In many of the studies, social aspect of TB is also underestimated. The men/women sex ratio shows a proportion much more important of men (3x) than women which suffers from the disease. Besides, the global drug resistance rates in men are twice higher than that of women. The 17–39 year group accounts for 80% of the studied strains, which shows that a young male population is specifically concerned by tuberculosis [1]. Contrary to the picture of most European countries, where the last years have seen a tremendous amount of studies describing the population-based genetic structure of *Mycobacterium tuberculosis*, no data of this kind are yet available for Turkey, with the exception of a recent paper describing the diversity of phospholipases genes in 106 clinical isolates of *M. tuberculosis *[2].

From a geographical, historical and anthropological aspect, Turkey is a link between Europe and Asia, an early region of human settlement, located in the Western part of Eurasia [3]. Various cultural and anthropological influences of early civilizations have left complex scars, making from Turkey an anthropologically rich area. More evidence about the unique nature and complexity of human genetics in Anatolia has been discovered, whether based on mitochondrial or on Y chromosome diversity [4-7]. The existence of a hypothetical proto-Indo-European language, whose link to Anatolia appears likely, is another issue that reinforces the importance of Turkey in human history, especially of the "Indo-European" lineages [8].

Molecular characterization studies on *M. tuberculosis *in Turkey until recently focused on the use of the "gold-standard" IS*6110*-RFLP method and on the description and characterization of multi-drug-resistance rates and their mechanisms, two issues of great importance for Public-Health [9-14]. However, to the best of our knowledge, no population-based data were available on the genetic diversity of *M. tuberculosis *in Turkey. The goal of this study was to get an initial insight into the biodiversity of *M. tuberculosis *in Turkey by studying a set of 245 DNAs from Anatolia by spoligotyping, a high throughput technique for which large polymorphism databases have been created [15]. These DNAs originated from as many clinical isolates from tuberculosis patients resident in Turkey. We show in this paper that a single genotype or "clonal complex" (ST41) accounts for one fourth of the total TB cases. A second genotype, ST284, may also bear some Eastern Mediterranean or Asian specificity but remains to be more exhaustively investigated. The predominance of a specific clonal complex in Turkey may argue in favour of a long-lasting presence of tuberculosis in this country and open new avenues of investigation to better understand why and how such a clone became predominant.

## Results

### Result of the genotyping analysis by spoligotyping

**Figure 1:**
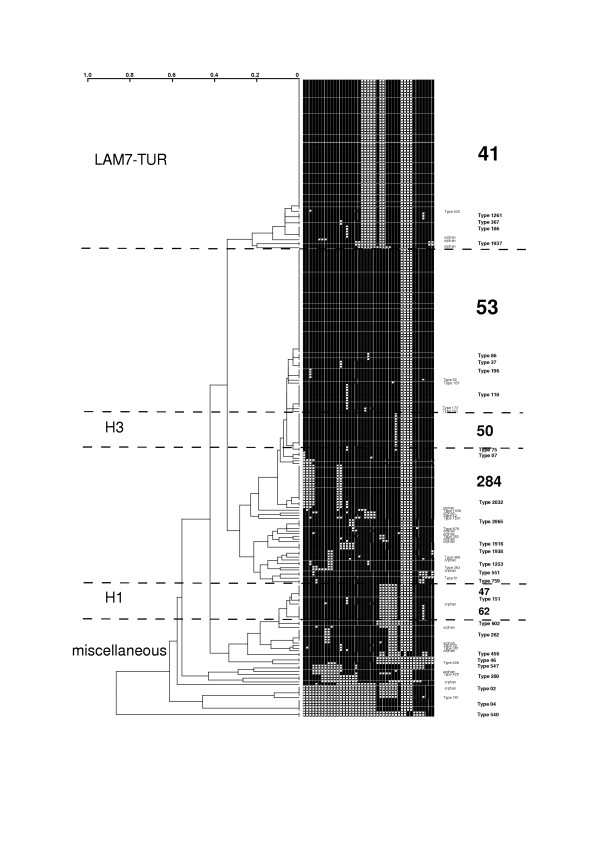
UPGMA tree constructed by numerical analysis of spoligotyping data using the Taxotron software (P.A.D. Grimont, Taxolab, Institut Pasteur, Paris). Remarkable genotypes are shown in bold at the extreme right of the picture (ST n°). The size of the characters is proportional to the frequency of genotypes. Dotted lines and some families (Haarlem1 and 3 as H1 and H3 and the newly "LAM7-TUR" family) are shown at the left of the dendrogram. Unique patterns are designated either as "orphan" or with their ST n° if already described in the SpolDB4 database.

The global structure of the *Mycobacterium tuberculosis *population by spoligotyping is shown on the Figure 1. Our results provide a first raw information on the distribution of the spoligotypes within two cities of Turkey. One spoligotype, ST41, is highly frequent. This spoligotype had already been detected in SpolDB3 and designated as LAM7, given the absence of spacer 21–24 in this superfamily of genotypes [16]. Another genotype, ST284 (frequent MIRU-VNTR : 223323153322), which remains undesignated for the time-being, is also frequent. We identified 37 different STs in the set of DNAs originating from Malatya (n = 147) and 42 genotypes in the set of DNAs originating in Ankara (n = 98). These results suggest that the genetic diversity in Ankara is superior to the one found in Malatya, which would be logical given that Ankara is a larger city and the administrative capital of Turkey. The data were further compared to the spolDB4 database [17]. Nine spoligotypes from Malatya were truly unique ("orphans") whereas 3 were in cluster with other clinical isolates found in the database: ST1936 was created by match with a clinical isolate from Sweden; ST 1937 (internal cluster) was created by a match between two clinical isolates T53 and T92, which are presumed to be linked to ST41; ST1938 was created by a match between two clinical isolates from Turkey (N500, N502) and one from Indonesia. The study shows that more than 58% of the patients in Malatya and 38% in Ankara, were gathered in only four different STs which are ST41 (LAM7-TUR family, 50/245 : 21%), ST53 (ill-defined T1 superfamily, 40/245 : 16.3%), ST50 (Haarlem 3 family, 13/245 : 5.3%), and ST284 (undesignated, 14/245 : 5.7%). It is likely that these tuberculosis genotypes are representative of strains that were introduced in Turkey at a distant past. If we compare the distribution of these genotypes between the rest of the world and Turkey, we note that ST41 prevails largely in Turkey (21% versus 0.35% in SpolDB4) whereas ST53 and ST50 are distributed equally world-wide and in Turkey. ST284 is poorly prevalent in the world database (0.1%) whereas highly frequent in our study, which also suggests a strong local phylogeographical specificity.

### Result of the genotypic analysis by IS6110-RFLP typing

**Figure 2:**
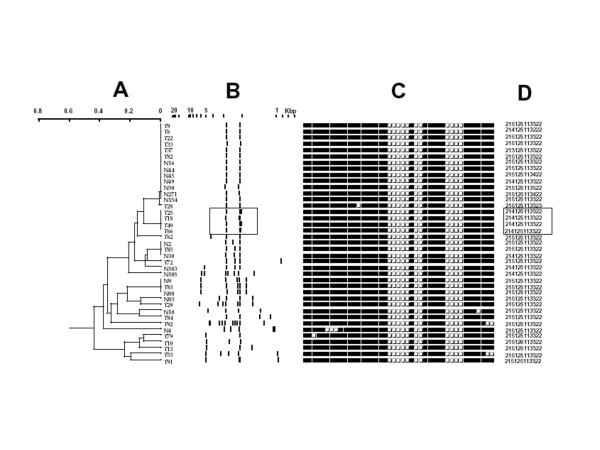
Partial UPGMA tree constructed by combined numerical analysis of IS*6110*-RFLP and spoligotyping data using Taxotron, and built on isolates bearing ST41 spoligotypes and variants. A: unrooted dendrogram with dissimilarity index scale, B: IS*6110*-RFLP patterns, C: spoligotyping pattern (binary representation), D: MIRU genotyping values for ST41 clinical isolates. Note the association of value 4 of MIRU10 and the IS*6110*-RFLP cluster defined by isolates T25, T18, T49, T66 and the observation of a double band at 2.2 kb.

The full results describing the complete molecular epidemiological analysis of the 145 clinical isolates, representative of as many patients from Malatya will be reported elsewhere. However, when we looked at the IS6*110*-RFLP results on the isolates bearing ST41, we confirmed that most of them harbored a similar IS*6110 *pattern based on a 2-copy signature, at around 2.1 kb and 2.8 kb respectively (Figure 2). Another subgroup of 5 clinical isolates harbored a slightly different 2-banded profile (2.1 and 4.8 kb; Figure 2). The description of TB genotypes harboring two copies of IS*6110 *was already reported in previous studies [13,18].

### Result of the genotypic analysis by MIRU-VNTR typing

A total of 33 clinical isolates belonging to the ST41 were further genotyped by MIRU-VNTR typing. Our aim was to apply this highly discriminant method to further distinguish between isolates with ST41 spoligotypes. Results are shown in Table 1. A main pattern, found in 19 clinical isolates was observed (215125113322). Another well represented variant pattern was found in 8 clinical isolates (214125113322). When a minimum parsimonious tree was built by minimum spanning tree, a unique clonal complex was observed (Figure 2).

### Result of other databases search

The 215125113322 pattern was introduced into the SITVIT1 database (spoligo-MIRU-VNTR international database, to be described elsewhere). Two isolates previously reported by L. Cowan (USA08096990178) and J. Driscoll (USA012004S00232) bearing the same ST41 spoligo and the same MIRU-VNTR pattern were identified. This MIRU-VNTR pattern was designated as VNTR-international-type (VIT) number 310. The second most represented allele 214125133322 bore the VIT number 194. When the clinical isolates bearing this MIRU-VNTR type value were compared to the IS*6110*-RFLP pattern of the same isolates (done in blind), an homogenous cluster of four fully identical IS*6110*-RFLPs with a double band at 2.2–2.8 kb was detected (T25, T18, T49, T66, cf. Figure 1 boxed), thus suggesting the full linkage between the two markers.

When a search was made against another database, the New-York state spoligotyping database, (version 2005 March 1^st^) maintained at the Wadworth Center in Albany, NY, we observed that ST41 (spoligopattern NYS_00232 in the NY database) was also found in 28 more clinical isolates in the USA, among which 22 were from Turkish clinical isolates from the city of Samsun, on the border of the Black Sea (J Driscoll and A Sanic, personal communication).

## Discussion

Tuberculosis remains a great public health concern in Turkey. The resistance to antituberculosis drugs, which represents a specific threat and as such deserves much attention, was recently the focus of many investigations in Turkey [9-14], however, molecular epidemiology is of more recent interest [13,18]. Indeed, the unraveling of the effect of genetic variability of *M. tuberculosis *on the presentation of the disease remains a challenging poorly investigated issue, which consists in understanding why a strain may become prevalent in certain communities [19]. Recent results obtained on the polymorphims of genes known to be involved in pathogenicity and virulence (phospholipases) may create a bridge between pathogenicity and population genetics studies [2]. However, the population-based genetic landscape of tuberculosis biodiversity was, to the best of our knowledge, unknown before this study and as such deserved such an attempt to define which genotypes are responsible of the TB cases in Turkey, a subject which, given the highly complex anthropological structure of Turkey, is of great interest for clinical scientists, bacteriologists, and evolutionary biologists.

A total of 245 DNAs extracted from *M. tuberculosis *clinical isolates from TB Turkish patients were genotyped by spoligotyping. A major genotype, as revealed by spoligotyping ST41, which misses spacer 20–24, 26–27 and 33–36, and had been previously described under the designation of "LAM7", represented up to one fourth of all TB isolates. When these genotypes were further investigated by IS*6110*-RFLP or by the highly discriminant MIRU-VNTR technique, highly similar profiles were obtained suggesting that these strains define a true genotype family or clonal complex. This genotype is likely to be identical to the one described in Table 2 by IS*6110*-RFLP and pTBN12 in a recent paper [13]. However, given the highly discriminative power of pTBN12 as a second genotyping method, and the difficulties to compare these patterns, a total of 7 subclusters were described initially (Ia to Ig). How these previous results correlate to MIRU-VNTR-based or spoligotyping-based clustering remains to be further investigated.

The finding of another genotype ST284 that was already detected in SpolDB3 but without origin of potential phylogeographical specificity is intriguing. This genotype is currently under investigation and is also found to be present in Bulgaria (T. Zozio *et al*., unpublished obervations). Whether this genotype also bears a larger Eastern-Mediterranean or Middle Eastern phylogeographical specificity remains speculative for the time-being.

The incidence of tuberculosis in Turkey was recently estimated around 26.6 new cases per 100.000 inhabitants [1]. For a city such as Malatya (853.658 inhabitants), which has a slightly superior incidence (32/100.000), the total estimated number of new cases per year would be n = 272. Thus, our sampling (n = 145) represents the equivalent of a quarter of a two-year recruitment, which, we assume, is fully representative of the genetic diversity in Malatya. In Ankara, for which the recruitment was less important, similar genotyping results were obtained. In a third city from the border of the Black Sea (Samsun), similar results were also obtained on 100 DNAs by an independent team (A. Sanic and J. Driscoll, personal communication). Thus the genotyping results obtained in Ankara and Malatya seems to be quite representative of Anatolia, suggesting that the ST41/VIT310 and ST41/VIT196 could represent traces of a contemporary and/or historically endemic/epidemic clone in Anatolia. If further investigations on isolates from the Aegean, Mediterranean and eastern sides of Turkey confirm the prevalence of the ST41 genotype, and provided that it is really ancient, one may expect that its distribution will vary depending on the human population structure in Turkey. Thus, the observed geographical variations in the frequency distribution of ST41 may allow to precisely define its presumed origin. However one should be extremely cautious with such historical inferences. Indeed, one should not forget that epidemics by their bursting nature, may rapidly promote the replacement of genotypes by others and that recent human migrations do complexify the issue [20]. Turkey is a country where ancient Central Asian and European civilizations can be seen. Preliminary phylogenetical results (not shown) suggest that the LAM7-TUR genotype family of *M. tuberculosis *may be related to the large LAM9 superfamily of genotypes; however, another spoligotype, ST353, could also be the ancestor type of ST41 and cannot be excluded at this step as a potential ancestor of ST41. Further studies using combined MIRU-VNTR-spoligotyping will facilitate the finding of the ancestor clone of the LAM7-TUR family.

## Conclusion

We described a new phylogeographically-specific clone of *M. tuberculosis*, designated LAM7-TUR. Further investigations to assess its frequency within all regions of Turkey and its phylogeographical origin and phylogenetic position within the global *M. tuberculosis *phylogenetic tree will shed new light on its endemicity in Asia Minor.

## Abbreviations

ST : Shared type, LAM : Latino-American and Mediterranean, MIRU-VNTR : Mycobacterial Interspersed Repetitive Unit-Variable Number of DNA Tandem Repeats, TUR : Turkey.

## Methods

### Studied Population

Turkey counted in 2003 more than 71 million inhabitants leaving in an urban zone (64,9 %) twice more than those leaving in a rural setting (35,1 %). In 2000 incidence of tuberculosis in Turkey was about 26,6 cases for 100000 inhabitants [2]. Sixty-four percent of the patients within this study were men (mean age = 35) in between 21 to 64 years (patient data from Malatya only, n = 147). The diagnostic of pulmonary tuberculosis was done for the 147 patients for whom 147 clinical isolates were identified. All the patients were resident in Malatya except for 2 individuals who lived in Adiyaman. The recruitment covers the 1998–2004 period.

### Spoligotyping

The Direct Repeat (DR) locus, which consists in multiple direct variable repeats (DVRs) [21] was assessed by the PCR-based reverse-line-blot nucleic acid hybridization method called "spacer oligonucleotide typing" or spoligotyping [22,23]. Spoligotyping was done at the Pasteur Institute of Guadeloupe using home-made membranes and results entered in Excel spreadsheets, Bionumerics (Applied Maths, Sint-Martens-Latem, Belgium), and Taxotron (PAD Grimont, Taxolab, Institut Pasteur, Paris).

### IS6110-RFLP

IS*6110*-Restriction-Fragment-Length-Polymorphism was done using the standardized method [24]. The genotyping was done on the Malatya's isolates only in Dr. Durmaz's laboratory in Turkey. Results were analyzed using H37Rv as international standard and comparison was done using Bionumerics (Applied Maths, Sint-Martens-Latem, Belgium). Strain H37Rv was used as the reference standard for IS*6110*-RFLP. Results were exported to Taxotron as a Molecular weight text file, a pairwise distance matrix was built using the Dice Index, and this file was summed and averaged to a similar pairwise distance matrix of the spoligotyping results built using the Jaccard Index, to produce the results shown in Figure 2 (combined numerical analysis, Taxotron's manual).

### MIRU-VNTR-typing (Mycobacterial-Interspersed-Repetitive-Units-Variable-Number of Tandem-Repeats typing)

MIRU-VNTRs were amplified from 12 genomic loci using 4 different multiplex PCRs with the previously described fluorescent primers, except that Hex labeling was replaced by Vic labeling [25]. Amplification was performed with HotStartTaq polymerase (Qiagen) using the same cycling conditions as in [25], except that 30 cycles were used instead of 40. Two μL of PCR products were mixed with 10 μL of formamide and 0.2 μL of MapMarker1000 ladder (bioventures). DNA fragments were separated by capillary electrophoresis using the ABI Prism 3100- Avant Genetic Analyzer (Applied Biosystems) as described in [26]. Sizing of the PCR fragments and assignment of the various MIRU-VNTR alleles were done using the GeneScan and customized Genotyper software packages (PE Applied Biosystem). MIRU-VNTR typing was done at the Pasteur Institute in Brussels.

### Phylogeny reconstruction: Taxotron (numerical taxonomy) and Bionumerics, Minimum Spanning Tree (population modeling)

**Figure 3:**
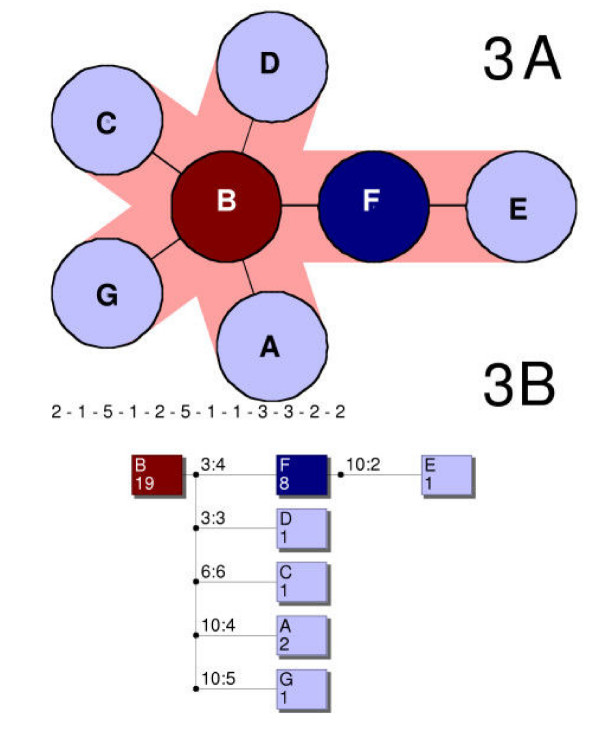
Minimum spanning tree built using the Bionumerics sofware on ST41-Mirutyped DNAs (Version 3.5, Applied Maths, Sint-Marteen-Latem, Belgium). 3A : tree panel window. 3B: complex panel window showing the MIRU value of the presumed origin of the tree. Genotypes are arbitrarily designated by letters. B : 215125113322 is suggested as the original genotype. For each designation, the number of cases is indicated (ex: 22 cases for genotype B). The number on the branch indicates the change required to go from one allele to another : exemple: a change on the 3^rd ^MIRU on genotype B from an allelic value = 5 to a value = 4 provides genotype F.

The Pairwise distance between clinical isolates was computed using the 1-Jaccard (1-*Sj*) index for the spoligotyping method [27] and using the Dice Index for IS*6110*-RFLP results [28]. The UPGMA algorithm (unweighted pair-group method using arithmetic averages) was used for clustering [29]. Distance-based methods are indeed fairly popular methods that have proved to be very useful to define some major phylogeographical clades within the *M. tuberculosis *complex [30,31]. The Bionumerics software (version 3.5) (Applied Maths, Sint Martens-Latem, Belgium) was used to reconstruct the hypothetical evolution of the ST41VIT310 clonal complex, following the user's manual (Figure 3).

### Database Search

A search was done using two Polymorphisms databases. SITVIT1 (which was designed and maintained at the Pasteur Institute of Guadeloupe, query done on March 1^st^, to be described elsewhere) and the New York State spoligotyping database (version 2005 March 1^st^), which was developed and maintained by Dr. J. Driscoll at the Wadsworth Centers.

## Authors' contributions

CS contacted ZS in Ankara and suggested a collaborative work. AA worked in collaboration with ZS to isolate and characterize the *M. tuberculosis *clinical isolates from the Ankara region, prepared quality DNA and send it to CS. To increase recruitment and representativity of the sampling, another collaborative work was simultaneously launched between NR and RD. RD and SG have isolated the strains from Malatya, characterized it, extracted the DNAs, done IS*6110*-RFLP typing in RD's laboratory and send DNAs to NR. TZ did the spoligotyping experiments of all the data shown here in NR's laboratory with auditing of the results by CS, at the Institut Pasteur of Guadeloupe. CA did the MIRU-VNTR-typing experiments in MF's laboratory in Brussels on DNAs aliquoted and sent by TZ. CS conceived the study, participated to its design, coordinated the action, interpreted the results with TZ and CA, built the figures and tables, wrote the paper with TZ.

## Figures and Tables

**Table 1:** MIRU results for 36 isolates with ST41

origin	clinical isolate n°	MIRU typing result	Octal spoligo result
Malatya	N2	215125113322	777777404760771
Malatya	N9	215125113322	777777404760771
Malatya	N16	215125113322	777777404760771
Malatya	N30	214125113322	777777404760771
Malatya	N44	215125113322	777777404760771
Malatya	N45	215125113422	777777404760771
Malatya	N49	214125113322	777777404760771
Malatya	N50	215125113522	777777404760771
Malatya	N83	215125113322	777777404760771
Malatya	N88	215125113322	777777404760771
Malatya	N154	215125113322	777777404760771
Malatya	N271	215125113422	777777404760771
Malatya	N503	214125113322	777777404760771
Malatya	N505	214125113322	777777404760771
Malatya	T6	214125113222	777777404760771
Malatya	T9	215125113322	777777404760771
Malatya	T10	215126113322	777777404760771
Malatya	T13	215125113322	777777404760771
Malatya	T18	214125113322	777777404760771
Malatya	T22	215125113322	777777404760771
Malatya	T25	214125113322	777777404760771
Malatya	T29	215125113322	777777404760771
Malatya	T33	215125113322	777777404760771
Malatya	T37	213125113322	777777404760771
Malatya	T49	214125113322	777777404760771
Malatya	T62	215125113322	777777404760771
Malatya	T66	214125113322	777777404760771
Malatya	T72	215125113322	777777404760771
Malatya	T82	215125113322	777777404760771
Malatya	T83	215125113322	777777404760771
Malatya	T84	215125113322	777777404760771
Malatya	T85	215125113322	777777404760771
Malatya	T91	215125113322	777777404760771
Ankara	1	215125113322	777777404760771
Ankara	7	215125113322	777777404760771
Ankara	8	215125113322	777777404760771
